# Ecosystem engineers in the extreme: The modest impact of marmots on vegetation cover and plant nitrogen and phosphorus content in a cold, extremely arid mountain environment

**DOI:** 10.1002/ece3.9948

**Published:** 2023-03-27

**Authors:** Piotr Chibowski, Marcin Zegarek, Aleksandra Zarzycka, Małgorzata Suska‐Malawska

**Affiliations:** ^1^ Faculty of Biology, Biological and Chemical Research Centre University of Warsaw Warsaw Poland; ^2^ Mammal Research Institute Polish Academy of Sciences Warsaw Poland

**Keywords:** arid region, burrow, Eastern Pamir, ecosystem engineering, extreme, long‐tailed marmot

## Abstract

Burrowing mammals strongly impact plant communities. One of the main effects is accelerating nutrient cycling and thus promoting plant growth. This mechanism is well‐studied in grasslands and alpine habitats, but less is known about this phenomenon in arid, cold mountain environments. We studied ecosystem engineering by long‐tailed marmots (*Marmota caudata*) by measuring the content of plant nitrogen and phosphorus, as well as nitrogen stable isotopes in plant biomass and marmot feces in a distance gradient up to 20 m from marmot burrows in an extremely arid glacier valley in Eastern Pamir, Tajikistan. We also captured aerial images of the area inhabited by marmots to study the spatial distribution of vegetation. There was a weak relationship between the presence of burrows and vegetation cover on soil not covered by burrow material. Burrow mounds were not colonized by plants, as opposed to other studies, where mounds are often microhabitats that enhance plant diversity. A significant increase in N and P in aboveground green plant biomass in the proximity of burrows was found in one out of six studied plant species. Contrary to our expectations, stable N isotopes did not give further insight into N routing. We assume that plant growth is strongly limited by water availability, which prevents them from utilizing the local increase in nutrients, certainly provided by marmot activity. The results are contrary to numerous studies, which showed that the role of burrowing animals as ecosystem engineers increases with increasing abiotic stress, including aridity. This shows a lack of this type of study at the end of the gradient of abiotic factors.

## Abstract 2

The presence of marmots and their burrows has surprisingly little effect on plant nutrient content and on vegetation cover in an extremely arid, cold mountain habitat. Burrow mounds are no microhabitats, as there is no plant succession on them. The results are contrary to previous studies on the effect size of ecosystem engineering in arid environments.

## 1 INTRODUCTION

Mammal species that build burrows and spend a part of their lives underground (hereafter called burrowing mammals) play an important role in their ecosystems (Beca et al., 2021; Davidson et al., 2012; Root‐Bernstein & Ebensperger, 2013), and have been described as keystone species (Whitford & Steinberger, 2010) and ecosystem engineers (Wesche et al., 2007). Burrows are often used as shelter by other vertebrates (Ewacha et al., 2016; Murdoch et al., 2009) and invertebrates (Sorokina & Pont, 2011; Yoshihara et al., 2010). Burrowing mammals can occur in high densities, therefore they also impact their environment through nonengineering effects (Prugh & Brashares, 2012) and can be important prey for predators (Davidson et al., 2012). But probably the most affected group of organisms are plants. Burrowing mammals expose soils through the forming of mounds of burrow material, consume plants and trample them, altering the competition between plants and promoting pioneer species. Additionally, mounds mainly consist of soil from beyond the surface, which has different chemical properties than surface soil. Thus, it promotes different plant species than in the surrounding area. The area around a burrow offers varied microhabitats and each can support a different plant community (Ballová et al., 2019; Sasaki et al., 2013). Hence, an increase in plant biodiversity and species richness on the landscape level can occur (Ballová et al., 2019; La et al., 2003; Lindtner et al., 2018; Niu et al., 2020; Valkó et al., 2020). Feces, urine, and animal carcasses (in the case of predators) increase soil nutrient content around the burrow. The vegetation around the burrow profits from this, increasing its nutritional status. In the proximity of burrows, the content of nitrogen and phosphorus in plant biomass is usually 20%–30% higher than in the surrounding area (Villarreal et al., 2008; Whicker & Detling, 1988), but some studies show an increase of up to 100% in N and 200% in P (Van Staalduinen & Werger, 2007). On the other hand, the positive effect on plant growth fades above some level of burrow density (Guo et al., 2012), as the high level of disturbance (grazing, digging, and trampling) results in the creation of bare soil patches with no vegetation, which are more prone to water evaporation and N loss (Pang et al., 2020). Therefore, the relationship between burrowing animal activity and vegetation cover can be manifold. In habitats where undisturbed areas are completely covered with vegetation, the presence of burrow entrances and mounds obviously decreases total vegetation cover (Louw et al., 2019). However, burrowing animals can also cause an increase in vegetation cover, but it is limited to a certain magnitude of disturbance, above which cover drops (Tang et al., 2019). This type of unimodal relationship is also found for the impact of burrowing animals on species richness or aboveground biomass (English & Bowers, 1994; Guo et al., 2012; Pang & Guo, 2017; Whicker & Detling, 1988) and is considered a classic example to support the intermediate disturbance hypothesis (Connell, 1978; Grime, 1973). Because of the reasons mentioned above, burrowing animals (mainly mammals) were often considered and treated as pests: an approach that has been criticized by scientists due to the need for biodiversity preservation and the role of burrowing species in the environment (Delibes‐Mateos et al., 2011; Smith & Foggin, 1999) but also because their negative impact on plant yield and pasture productivity is doubtful (Bagchi et al., 2006; Guričeva, 1985).

Studies on the impact of burrowing animals on vegetation are numerous, but the majority of them were conducted on steppes, arid and semi‐arid plains, including pastures or alpine meadows, as well as hot deserts (Beca et al., 2021; Davidson et al., 2012; Mallen‐Cooper et al., 2019; Platt et al., 2016; Root‐Bernstein & Ebensperger, 2013). Almost no studies show the ecological roles of burrowing mammals in extremely arid high‐altitude habitats in an early stage of soil formation and plant succession, where plant productivity is limited by water shortage and low temperatures, rather than nutrient availability and competition for space. High‐altitude habitats are among the regions where climate change is the most rapid (Kohler et al., 2010). Melting glaciers are exposing mineral soils, which are slowly being colonized by microorganisms and plants (Egli et al., 2012). Whether this process can be facilitated by burrowing ecosystem engineers is unknown. The first step to answering this question should be a study of the impact of burrowing mammals on plant communities already established in glacier forelands.

We studied the impact of the activity of long‐tailed marmots (*Marmota caudata*, hereafter referred to as marmots, as they are the only species from this genus in the study area) on selected plant parameters. The long‐tailed marmot is a burrow‐dwelling rodent that inhabits a large part of Central Asia. It is a large rodent, typically between 1.5 and 7.3 kg heavy, and is known to occupy a wider range of habitats than related marmot species, occurring from 600 to 5200 m a.s.l and being relatively tolerant of aridity (Krystufek & Vohralik, 2013). Long‐tailed marmots are herbivores consuming the green parts of plants and foraging closely to the entrance of their burrow. They hibernate from September to April – May (Blumstein & Arnold, 1998). Marmots are generally attached to their main burrows, which can be used for several generations (Barash, 1989). Apart from the main burrow system, which can consist of extensive corridor systems and multiple entrances, they also build shallow, single‐entrance escape burrows (Blumstein, 1998).

The study was done in an extremely dry habitat in Eastern Pamir with hampered soil development and plant succession (Kabala et al., 2021). The mechanisms that we aimed to study were the role of the mound as a microhabitat and the combined impact of excretion and herbivory on plant cover and nutrients, which can be considered a proxy for plant fitness and habitat suitability. Apart from that, we report some characteristics of the distribution of burrows, which we recorded during our study. The species is largely unstudied and those results might be found useful. We used an unmanned aerial vehicle to take images of the area and map vegetation. We measured N and P content in aboveground green plant biomass in a distance gradient from burrow entrances, to check whether plants are utilizing the nutrient input from marmot feces. We expected large species‐specific differences in nutrient content in plant biomass caused by different adaptation strategies to low temperatures (Körner, 2003) and therefore measured each plant species separately, instead of mixing all aboveground biomass. We used stable isotope composition as a marker of animal‐derived N. Preliminary field observations of vegetation in the study area suggested, that the impact of marmots on plants is not pronounced, as there was no visible change in vegetation properties around burrows (Suska‐Malawska M., Zegarek M., Sulwiński M., unpubl.), unlike in many other studies (Ballová et al., 2019; Fafard et al., 2019). Also, feces were seen mainly on mounds, close to the burrow entrance. Therefore, we planned our study on a small scale, in 20‐m buffers around burrow entrances. We did not collect control samples, due to difficulties in planning proper sampling (see Appendix S1 for a detailed explanation). Our goal was to conduct a descriptive study of a well‐known mechanism in previously unstudied conditions.

## 2 MATERIALS AND METHODS

Burrow survey was done in July 2018. Sample collection, aerial images, and additional burrow search were done in July 2019.

### 2.1 Study area

**FIGURE 1:**
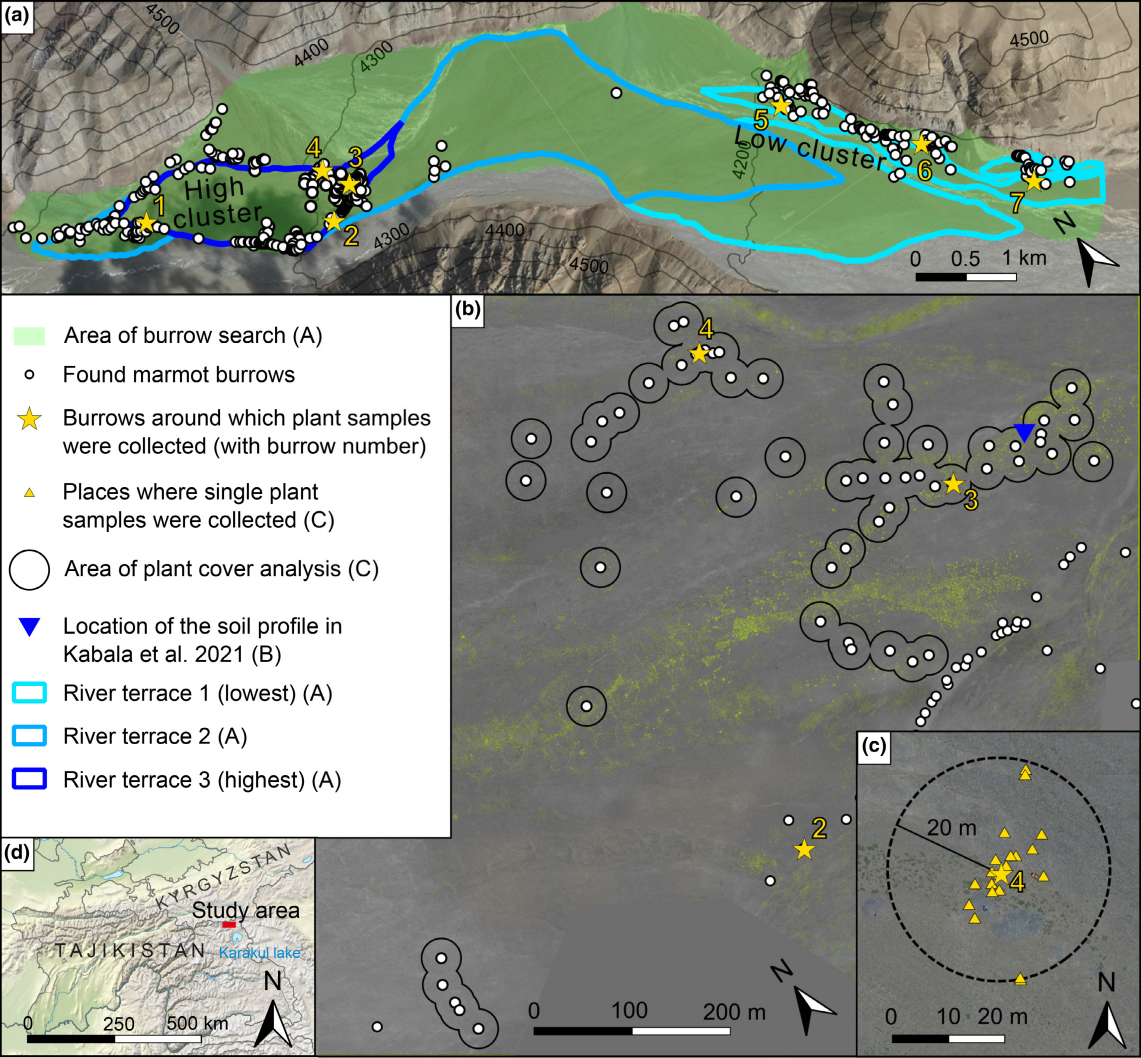
The study area in the North‐Western part of the Koksoy river valley. (a) Overview of the study area, location of burrows, search area, and sampling sites with Google satellite images. (b) Area covered by aerial imagery with 20 m buffers around analyzed burrows and burrow aggregations. The green channel is enhanced, so vegetation is visible. Note, that this is not an exact visualization of plant occurrence, as it was not manually corrected and can be biased by varying lighting conditions in different parts of the image. (c) Example of sampling spots of plant biomass around a burrow with 20 m buffer. (d) Location of the study area in Tajikistan.

**FIGURE 2:**
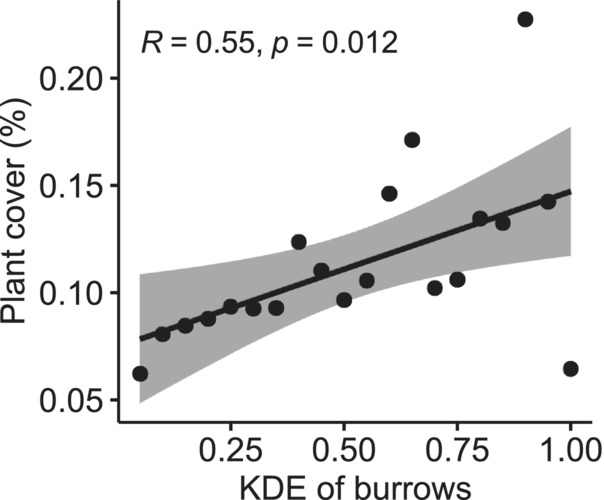
The relationship between the interval of kernel density estimate (KDE) of burrows and the plant cover.

The Koksoy river valley is located in Eastern Pamir in Tajikistan (39°19′15″ N, 73°13′32″ E) at altitudes between 4100 and 4400 m a.s.l. The valley is ca. 16‐km‐long, up to 1.5‐km‐wide (Figure 1a) and oriented west‐northwest, with the river flowing along the southern slope. Monthly average air temperatures in the valley are between −20°C in January and 5°C in July and August (Kabala et al., 2021). The mean annual precipitation in the nearest meteorological station close to Lake Karakul is ca. 80 mm (Mischke et al., 2010). Previous research showed that soil development and plant succession in this area are extremely slow, and organic soil carbon pools are among the lowest reported in glacier forelands (Kabala et al., 2021). Marmot burrows are concentrated in two locations: (1) on the highest out of three river terraces, which has an area of about 1 km^2^, and on an old alluvial fen adjacent to the terrace. Burrows are located mainly along the northern edge of the floodplain between 1 and 3 km from the glacier's front (hereafter called the high cluster, see Figure 1a); (2) along the northern slope of the valley, at a length of about 2.6 km, approximately in the middle of the valley, between 6 and 8.4 km from the glacier terminus. Burrows are located on flat ground on the lowest river terrace and the slope of the valley (hereafter called the low cluster, see Figure 1a). The soil in both locations is very poorly developed, but there are some differences in the upper layer (0–10 cm), caused by different ages of the river terraces. The soil on the highest, oldest terrace where the high cluster is located has a lower skeleton content and a higher silt, sand, and clay content than in the location of the low cluster. It also has higher total N and P (in Mehlich‐3 extract) content than the soil on the lowest terrace. Vegetation in the valley is sparse. In the area of the high cluster, cushion plants and perennial forbs dominate, and the average vegetation cover is 41.2%. Dwarf shrubs, mainly *Krascheninnikovia ceratoides* and cushion plants dominate in the area of the low cluster, and the average vegetation cover is 21.4% (Kabala et al., 2021).

### 2.2 Field inspection and burrow search

During the field inspection, we searched the main part of the valley—approximately 17.18 km^2^ (Figure 1a)—for marmot burrows and recorded their location using a GPS device. Burrows were visible from a long distance due to the distant color of burrow mounds. Our main goal was to find all burrows within the two clusters, which were identified during preliminary work. The remaining part of the valley was roughly searched by foot or by car. We treated every entrance as a separate burrow unless several entrance corridors were clearly merging into one corridor belowground. We noted the presence or absence of plants on the mound. A common method to check the activity status of a burrow is looking for fresh feces (Karels et al., 2004). We suspected, that due to the type of environment fresh feces dry out very quickly but then remain intact for a long time. Therefore, we counted all burrows, which had any feces on them. Burrows without feces were usually damaged. We considered them abandoned and did not record them.

### 2.3 Aerial images

We used an unmanned aerial vehicle (DJI Phantom 3 advanced) to capture aerial images of the main part of the high cluster (Figure 1b). Images were captured in similar lighting conditions, at 45 m above ground, with ISO speed 100, F‐stop 4.5, autoshutter, and white balance set as cloudy. The images were processed using Agisoft Metashape Professional v.1.5.5 and converted into an orthophoto mosaic with a 4‐cm pixel size. Due to long periods of strong wind, we did not manage to take photographs of the low cluster.

### 2.4 Collection of plant biomass and fecal pellets

For the analysis of nutrients and stable isotopes, we selected 7 burrows (4 burrows from the high cluster and 3 from the low cluster) with visible signs of recent marmot presence (fresh feces) to collect plant biomass samples and fecal pellets. We numbered the selected burrows from 1 to 7 (Figure 1a). To exclude the possibility of cumulative influence of burrows, we chose burrows that were further than 50 m from the entrance of the closest burrow with fresh feces. Around each burrow, we selected 2–4 (depending on the number and abundance of plant species) dominant plant species/genera, and we collected about 10 samples of aboveground green biomass from each (Table 1). Some samples got lost during transport, therefore the number is not always 10. Samples were collected at different distances from the burrow entrance, up to 20 m (Figure 1c). We collected 171 biomass samples from 8 plant species/genera in 20 combinations of burrow and plant. From each mound of the selected burrows, we collected 10 randomly selected fresh fecal pellets.

**TABLE 1:** Plant species collected for nutrient analysis, total number of samples, burrows at which the species were collected (number of samples in brackets), and the content of N and P and stable nitrogen isotope ratios (mean ± SD).

Plant species/genus	*N*	Burrows (*n*)	Total N (%)	Total P (%)	N:P	δ^15^N (‰)
*Acantholimon* sp.^1^ ^,a^	33	1 (10), 3 (9), 7 (9), 6 (5)	2.7 ± 0.31^c,e,f,g,h^	0.09 ± 0.03^c,e,f,g,h^	32.46 ± 11.94^c,d,e,f,g^	1.12 ± 1.71^g^
*Androsace* sp.^2^ ^,b^	6	2 (6)	1.99 ± 0.3^b,e,f,g,h^	0.07 ± 0.02^c,e,f,g,h^	31.38 ± 6.43^c,d,e,f^	−0.81 ± 0.56^f^
*Braya pamirica* ^c^	12	2 (6), 5 (6)	3.85 ± 0.79^a,b,e^	0.22 ± 0.07^a,b^	18.54 ± 4.68^a,b^	1.04 ± 1.93^g^
*Ephedra* sp.^3^ ^,d^	5	2 (5)	2.89 ± 0.35^e,f,h^	0.21 ± 0.12	16.41 ± 4.31^a,b^	1.11 ± 0.48
*Erysimum altaicum* ^e^	19	3 (9), 4 (10)	4.98 ± 0.88^a,b,c,d,f,g^	0.28 ± 0.12^a,b^	19.91 ± 5.84^a,b^	1.51 ± 2.38^g^
*Krascheninnikovia ceratoides* ^f^	30	7 (10), 5 (10), 6 (10)	4.05 ± 0.69^a,b,e^	0.23 ± 0.07^a,b,g^	18.31 ± 4.2^a,b,h^	1.87 ± 2.1^b,g^
*Oxytropis* sp.^4^ ^,g^	46	2 (12), 3 (9), 4 (10), 5 (10), 6 (5)	3.68 ± 0.54^a,b,e,h^	0.17 ± 0.04^a,b,e,f^	22.03 ± 3.55^a^	−1.01 ± 1.22^a,c,e,f,h^
*Leiospora eriocalyx* ^h^	20	1 (10), 5 (10)	4.44 ± 0.52^a,b,d,g^	0.18 ± 0.07^a,b^	26.04 ± 6.54^c,f^	1.05 ± 1.88^g^
		*H*	105.48	98.79	68.27	65.83
		df	7	7	7	7
		*p*	<.0001	<.0001	<.0001	<.0001

*Note*: Kruskal–Wallis test results are given for the comparisons between species. Letters next to values indicate plant species, which differed significantly based on pairwise comparison using the Dunn's all‐pairs test with Holm adjustment of *p*‐values.

To our best knowledge, the species within genera are (Chachulski unpubl.):

^1^ There was only one species from genus Acantholimon, probably *Acantholimon hedinii* Ostenf.

^2^ There was only one species from genus Androsace, probably *Androsace akbaitalensis* Derganc.

^3^ There was only one species from genus Ephedra, probably *Ephedra fedtschenkoae* Paulsen.

^4^ The collected individuals were probably *Oxytropis immersa* (Baker) Bunge ex Lipsky. Other species were present: *Oxytropis crassiuscula* Boriss, on the high cluster, and *Oxytropis poncinsii* Franch, on the low cluster, but both are much less abundant than *O. immersa*.

### 2.5 Laboratory analysis

Plant and fecal samples were air‐dried on the spot, and later oven‐dried at 40°C for 24 h. Plant biomass samples were then milled to a fine powder in liquid nitrogen using a pestle and mortar. From each burrow area, we randomly selected 3 sets of 3 fecal pellets. We milled each fecal pellet set together in liquid nitrogen to form 3 mixed samples from each burrow area. From each sample (plant or feces) we weighted approx. 5 mg into a tin capsule and measured total N (expressed as % of dry mass) using a Flash 2000 Elemental Analyzer. To measure P content approx. 100 mg of each sample (plant or feces) was microwave digested in nitric acid. The P content was measured using the molybdate method on a continuous flow San++ Skalar analyzer. Between 1 and 3 mg of each plant or feces sample (depending on N content) was weighed into a tin capsule and measured for nitrogen stable isotope composition using a Delta V Plus Isotope Ratio Mass Spectrometer, coupled with a Flash 2000 Elemental Analyzer via continuous flow. Stable isotope ratios were expressed as *δ*, i.e., as the deviation in per mille (‰) from the international standard, atmospheric nitrogen, in line with the equation: *δ*_sample_ = (*R*_sample_/*R*_standard_ − 1) × 1000, where *R* is the isotopic ratio, ^15^N/^14^N. International standards were measured for calibration and measurement precision, which was <0.1‰. All chemical laboratory analyses were done in the Laboratory of Biogeochemistry and Environmental Protection, Biological and Chemical Research Centre, University of Warsaw.

### 2.6 Data analysis

It was difficult to calculate the burrow density, as the area is a mosaic of different geological forms (Kabala et al., 2021). Some parts of the valley could be unsuitable for burrow digging or avoided by marmots for other reasons, affecting burrow density. Therefore, we had to determine the area for burrow density measurement arbitrarily.

We excluded burrows that were located on the edge of the highest river terrace from spatial analysis of aerial images. For the remaining 58 burrows, which were covered by aerial photographs, we analyzed land cover in round buffers with a radius of 20 m around the burrow entrance, resulting in an area of 5.29 ha. We divided the analyzed area into hexagons with a short diagonal of 25 cm, corresponding to an area of 0.054 m^2^. We assigned each hexagon to one of four categories: burrow entrance, burrow mound, bare soil or vegetation. The categories reflected the dominant type of cover (>50% area) within the hexagon. There was only one burrow entrance hexagon per burrow. Burrow mounds were easily distinguishable due to their gray color and determined through visual inspection of the orthophoto mosaic. Vegetation was identified using raster analysis with the Red‐Green‐Blue Vegetation Index (Bendig et al., 2015). All hexagons that did not fall in the two abovementioned categories were described as bare soil. Distance measurements were done between centroids of hexagons. For each buffer around a burrow, we measured the total vegetation cover of the area free of mounds as: vegetation hexagons/(vegetation hexagons + bare soil hexagons) and the area of the mound. For each hexagon (using the hexagon centre for measurement), we calculated the Kernel Density Estimation (KDE) of burrows, using a 20‐m radius and quartic function. KDE was scaled from 0 to 1, where 1 was the highest value found in this analysis. We then calculated the mean KDE of hexagons categorized as plant and soil. We divided hexagons into 20 intervals, based on KDE. For each interval, we calculated the total vegetation cover of the area free of mounds as: vegetation hexagons/(vegetation hexagons + bare soil hexagons). We plotted the vegetation cover of each interval against KDE and calculated the Pearson correlation coefficient (*R*).

Before statistical analysis of the results of chemical analyses, we checked whether the data met the requirements of parametric tests: normal distribution (using the Shapiro–Wilk test) and equality of variances (using the *F*‐test for two groups and the Bartlett test for multiple groups). We performed the Kruskal–Wallis test to compare the chemical parameters of plant species and the post‐hoc Dunn's all‐pairs test with Holm adjustment of *p*‐values. We used the two‐sided *t*‐test (for normally distributed data with equal variances), the Welch's *t*‐test (for normally distributed data with unequal variances), or the Wilcoxon signed‐rank test (for non‐normally distributed data) to check for differences in the abovementioned parameters between plants of the same species from the high and low cluster, and between feces from the high and low cluster. We calculated the Spearman's rank correlation coefficient (*ρ*) between the measured parameters in biomass and the distance of the plant from the burrow entrance, separately for each plant species. Based on N and P content in plant biomass, we calculated the N:P ratio.

We used QGIS v. 3.10.14 software for spatial analysis (QGIS Development Team) and map preparation, and R v.4.0.5 software (R Core Team, 2021) for statistical analysis, using the build‐in “stats” package. We used the “ggplot2” package (Wickham, 2016) to prepare graphs.

## 3 RESULTS

### 3.1 Burrow survey

We found 374 burrows: 241 within the high cluster, 127 within the low cluster, and 6 in other locations around the valley (Figure 1a). The burrow density in the whole searched area was 0.22 burrows/ha, on the high cluster 0.82 burrows/ha and on the low cluster 0.97 burrows/ha. Most marmot feces were placed in stacks of several pellets on the mounds, only seldom in the surrounding area. We found no signs of plant succession on any burrow mound.

### 3.2 Spatial data from aerial images

The area of mounds varied between 0.87 and 82.81 m^2^ (mean 25.21 ± 17.80). The total vegetation cover in a 20 m buffer around a burrow entrance varied between 0.27 and 34.64% (mean 8.63 ± 8.42%). Hexagons categorized as plants were at higher burrow KDE values (0.15 ± 0.13) than bare soil (0.13 ± 0.12). Plant cover was positively correlated with burrow density (*R* = .55, *p* < .05, Figure 2).

### 3.3 Chemical parameters of aboveground plant biomass

**FIGURE 3:**
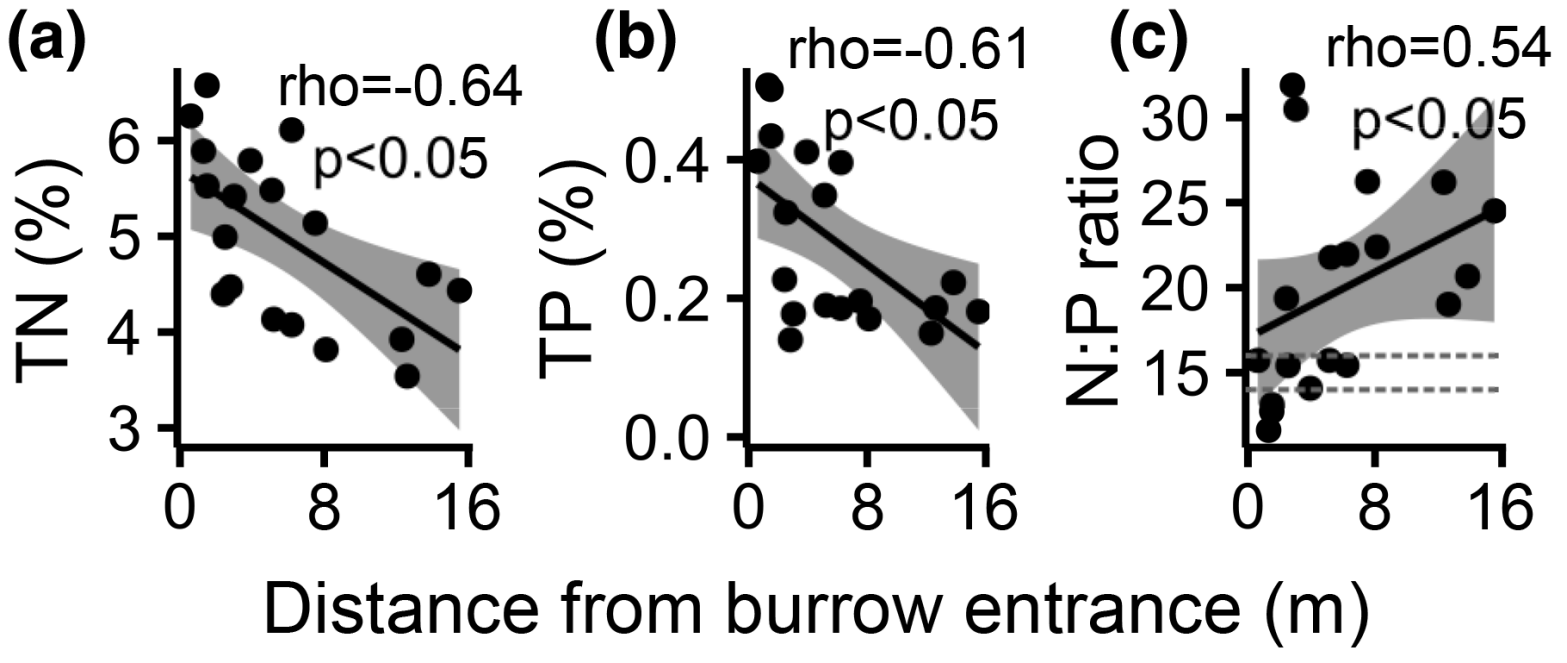
The relationship between the distance from the burrow entrance and parameters of plant samples of a given species. (a) Total nitrogen (TN), (b) total phosphorus (TP), (c) N:P ratio. Only pairs with significant relationships are shown—for *Erysimum altaicum*. Plant species and Spearman's *ρ* are given. The dashed lines in the N:P plot indicate values 14, below which N limitation can be assumed and 16, above which P limitation can be assumed. See the discussion for details on the constraints of this inference.

Plant species differed significantly in all parameters which were measured in aboveground biomass (Table 1). *Acantholimon* sp. and *Androsace* sp. had the lowest N (2.70 ± 0.31% and 1.99 ± 0.30%, respectively) and P (0.09 ± 0.03% and 0.07 ± 0.02%, respectively) content, and the highest N:P ratios (32.46 ± 11.94 and 31.38 ± 6.43, respectively). *Erysimum altaicum* had the highest content of both N (4.98 ± 0.88%) and P (0.28 ± 0.12%). The high and low clusters varied in several measured parameters, depending on the plant species (Table S1). Most notably, *Braya pamirica* and *Oxytropis* sp. had higher N and P content in the high cluster than in the low cluster. Most parameters in most species were not significantly correlated with the distance from the burrow entrance (Table 2). We found significant correlations in *Erysimum altaicum* with total N (*ρ* = −.64, *S* = 1872.6, *p* < .05), total P (*ρ* = −.61, *S* = 1839.6, *p* < .05) content, and N:P ratio (*ρ* = .54, *S* = 521.46, *p* < .05; Figure 3).

**TABLE 2:** Spearman's rank correlation coefficient (*ρ*) between the distance from the burrow entrance and all measured parameters in plant biomass.

Plant species	Total N (%)	Total P (%)	N:P	δ^15^N (‰)
*Acantholimon* sp.	−.13	−.12	.07	−.16
*B. pamirica*	−.37	−.06	−.16	.23
*E. altaicum*	**−.64**	**−.61**	**.54**	−.02
*K. ceratoides*	−.07	−.14	.05	−.29
*Oxytropis* sp.	−.11	−.18	.16	.09
*L. eriocalyx*	−.16	−.3	.23	−.14

*Note*: Bold values indicate significant correlations (*p* < .05). Plants with a sample number below 10 (*Androsace* sp., *Ephedra* sp.), were excluded from the analysis. See Table 1 for full species names.

**FIGURE 4:**
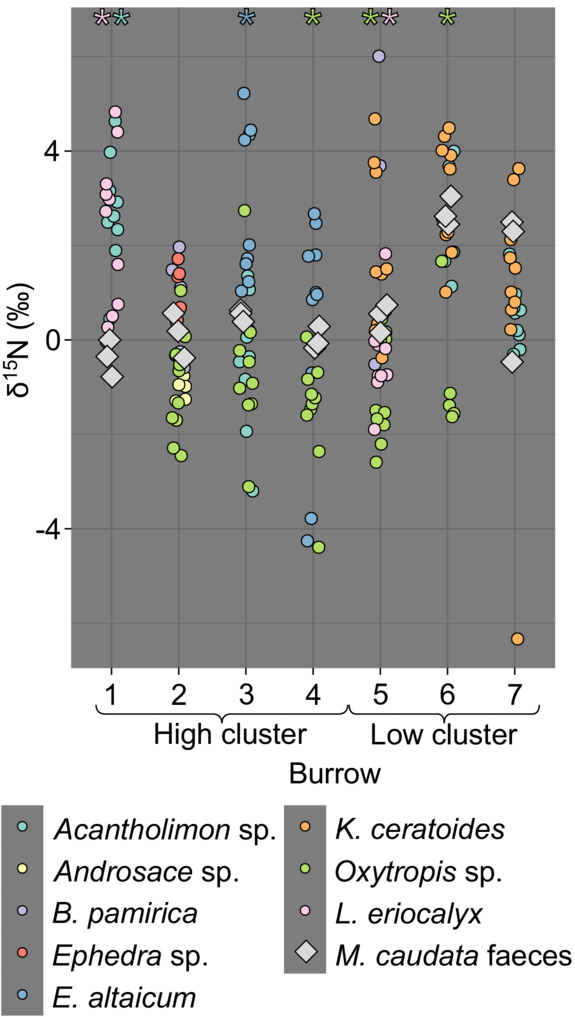
Stable nitrogen isotope ratios of plants and marmot feces collected on each burrow. Asterisks indicate significant differences between δ^15^N values of feces and those of a given plant species sampled from the same burrow, based on the Wilcoxon test (*p* < .05). The color of the asterisk indicates the plant species.

Feces from the high cluster had a higher content of N (4.2% ± 0.28) and lower δ^15^N values (0.07‰ ± 0.42) than feces from the low cluster (3.03% ± 0.71 and 1.54‰ ± 1.22, respectively). The content of P in feces was similar in both locations (0.7% ± 0.17 and 0.65 ± 0.19 on the high and low clusters, respectively). δ^15^N of plants and feces varied between −6.33‰ and 6.00‰. Out of 20 feces–plant species pairs, the δ^15^N values differed between plants and feces in 7 pairs (Figure 4). *Acantholimon* sp. (2.44‰ ± 1.36) and *Leiospora eriocalyx* (2.44‰ ± 1.52) had higher δ^15^N values than feces (−0.38‰ ± 0.32) on burrow 1 (*W* = 1, *p* < .0  and *W* = 0, *p* < .05, respectively), *E. altaicum* (2.87‰ ± 1.55) had higher δ^15^N values than feces (0.53‰ ± 0.1) on burrow 3 (*W* = 0, *p* < .05), *Oxytropis* sp. had lower δ^15^N values than feces on burrows 4 (−1.5‰ ± 1.13 vs. 0.01‰ ± 0.2, *W* = 28, *p* < .05), 5 (−1.22‰ ± 1 vs. 0.48‰ ± 0.24, *W* = 28, *p* < .05) and 6 (−0.81‰ ± 1.25 vs. 2.7‰ ± 0.25, *W* = 15, *p* < .05), and *L. eriocalyx* (−0.35‰ ± 0.91) had lower δ^15^N values than feces (0.48‰ ± 0.24) on burrow 5 (*W* = 27, *p* < .05).

## 4 DISCUSSION

We found surprisingly little impact of marmots on the studied aspects of plant life, with one puzzling exception of *E. altaicum*, which seems to utilize the additional nutrients provided by marmot feces. It is generally believed that the role of ecosystem engineers is more pronounced in harsh abiotic factors (Crain & Bertness, 2006). Below, we try to explain why our results failed to fit in this concept.

We found that plants grow denser in areas closer to one or several burrows during statistical analyses, despite not seeing such a relationship in the field or initially on aerial photographs. However, the effect size is not great, compared with some studies, where burrowing animals were shown to facilitate plant growth on nearly bare soil (Gharajehdaghipour et al., 2016). The large variation of plant cover in the surroundings of burrows (from close to no plants to over 30%) implies, that there is another driver of plant distribution. Field observations suggest that dense vegetation patches occur in shallow, merely visible channels on the surface of the terrace, which may seasonally carry meltwater. Given the limited effect of the additional nutrients on plants (see below), the slow growth of plants, and the fact that marmots graze almost exclusively in the burrow area (Blumstein, 1998), it is puzzling that vegetation cover does not seem negatively impacted by burrow proximity. We cannot explain this result, as we do not know the primary production of plants, the size of the marmot population and their feeding requirements, as well as the time spent in and around different burrows. The only study on the impact of long‐tailed marmot (or any other burrow‐dwelling species) activity on plants conducted in a comparable environment in Eastern Pamir, found a small impact of the presence of marmot burrows on plant species composition, and the results varied among study sites. Generally, dwarf shrubs occurred more often in disturbed areas than in undisturbed areas, as opposed to herbs (Dotter, 2009), which suggest that browsing does impact the plant community, as shrubs are more resistant to herbivory.

Our results on the relationship between the burrow density and N and P content in plant biomass are ambiguous. Only one out of six species, *E. altaicum*, showed an increase in biomass N and P content with increasing proximity of the burrow entrance. The effect size in this species is enormous, with both N and P content increasing significantly with burrow density and proximity. This implies that there is a nutrient input caused by marmots, and so do the result of analyses of soil sampled within the high cluster by Kabala et al. (2021). It had almost 100 times higher P (0.31 mg/kg) and 10 times higher N (0.169%) content than from other parts of the valley (0.47–0.125 mg/kg and 0.01%–0.06%, respectively), which the authors of this study attribute to marmot activity, as the sample was collected within the high cluster, close to several burrows (Figure 1b). This allows us to treat the mound area as a natural fertilization experiment and interpret it as such, although with necessary caution. The limited impact of nutrient addition on vegetation cover and biomass N and P content suggests, that most plant species are not limited by nutrient availability but probably by abiotic factors, mainly water scarcity, and they simply lack the necessary moisture to utilize the additional nutrients (He & Dijkstra, 2014). The effect size of nitrogen addition on plant nutrients was proven to be positively correlated with precipitation (Yahdjian et al., 2011). Burrowing animals are known to increase water availability for plants, as their constructions promote water infiltration and prevent water runoff and evaporation (Laundre, 1993; Whitford & Kay, 1999). However, this probably does not occur in our study area. Most of the precipitation is presumably instantly lost to evaporation, irrespective of the presence or absence of marmots. In turn, plants are adapted to these conditions and have deep roots, up to 50 cm (Kabala et al., 2021), to utilize any soil moisture. Even if there was an increase in water infiltration in the surface layer of the soil, it could probably not be used by plants. The response of *E. altaicum* to the marmot‐derived nutrient input implies that this species, apart from being capable to utilize the additional N and P, is more efficient at water uptake and/or preventing water loss than other species. However, we lack information about the functional traits of plant species from our study, so we are not able to confirm this hypothesis.

The results of N:P stoichiometry in the distance gradient from the burrow entrance show a shift from P limitation at higher distances to N limitation close to the entrance. The use of the N:P ratio to identify the limiting nutrient, where N:P < 14 indicates N limitation, N:P < 16 P limitation, and values in between indicate co‐limitation has been prosed for wetlands (Koerselman & Meuleman, 1996) and proven useful in grasslands (Craine & Jackson, 2010; Zhao et al., 2017). We are using this tool with caution because the high N concertation in plants inhabiting alpine habitats, and the resulting high N:P ratios (such as those we found in most species) are mainly caused by slow growth and adaptations to low temperatures, not by P limitation (Körner, 2003). Those adaptations are species‐specific and most probably site‐specific, therefore we are only looking at values within *E. altaicum*. They indicate that feces are a more important source of P than N. Feces have similar N content as plant biomass, whereas their P content is almost three times higher, indicating that they are a better source of P than plant litter. Soil P content can be also increased by the presence of marmot bones, which we have observed on several mounds. Up to 30% of marmots fail to survive the hibernation period (Blumstein & Arnold, 1998), their remains are probably ejected on the surface after hibernation or during burrow maintenance. Plants growing on initial soils are usually not P‐limited, as it is abundant from weathering minerals, as opposed to N (Walker & Syers, 1976). However, in high mountain continental areas, the low temperature and precipitation can hamper weathering to the point that plans are limited by P shortage (Darcy et al., 2018).

Increased N and P content in plant biomass close to burrows reported in the literature are mostly caused by the input of organic matter by the burrowing animal, but it can be also an indirect effect of animal activity: (1) Disturbed soil has higher temperatures and is more susceptible to N mineralization, increasing the amount of plant‐available soil N (Whicker & Detling, 1988); (2) Defoliation (including grazing) may increase N uptake by plants (Jaramillo & Detling, 1988; Yan & Lu, 2020). Therefore, we decided to use stable isotope composition as a marker of animal‐derived N used by plants. Feces of rodents are enriched in ^15^N compared with diet by 1.4‰–2.5‰ (Hwang et al., 2007; Sare et al., 2005) due to their discrimination in metabolic pathways. Given that long‐tailed marmots mainly stay close to the burrow while foraging (Blumstein, 1998), we hypothesized, that the elevated δ^15^N values in feces and the enhanced N cycling in burrow proximity would result in higher δ^15^N in plants growing closer to the burrow entrance. The lack of difference between δ^15^N values of plants and feces in most cases implies little isotopic enrichment of the latter. Results on isotopic enrichment (trophic discrimination) in feces are based on feeding experiments, where food is usually provided ad libitum. Fasting and nutritional stress, which is frequent in the natural environment, is known to affect δ^15^N values of animal tissues (Doi et al., 2017; Hertz et al., 2015) and could also alter the metabolic pathways leading to feces enrichment in ^15^N. Previous studies found no clear pattern of stable isotope ratios in the diet and feces of burrowing animals. Plains vizcacha (*Lagostomus maximus*) feces had lower δ^15^N values by approx. 4‰ than soils and shrubs at burrow systems, with no interpretation by authors (Villarreal et al., 2008). Grasses close to arctic fox (*Vulpes lagopus*) dens had similar δ^15^N values to grasses from control sites, despite the significant impact of fox presence on the available N pool in soils (Gharajehdaghipour et al., 2016). Authors attributed the loss of animal‐derived ^15^N to discrimination during mineralization and plant uptake (where high N availability could further increase discrimination). Other studies showed an increase in δ^15^N in plants around burrows, proving the fertilizing effect of animal feces (Ben‐David et al., 1998; García et al., 2002). However, those studies were done in burrows of predators, which have higher δ^15^N values in tissues and feces due to longer trophic chains (Fry, 2006). In such a case, when groups involved in the relationship are separated by several trophic steps, each with its specific isotopic enrichment (Vander Zanden & Rasmussen, 2001), the isotopic difference and the resulting method resolution is high enough.

A mechanism typical for the interaction between burrow‐dwelling animals and plants, which does not occur under the circumstances we studied is the role of burrow mounds. They consist of subsurface soil and are initially bare, creating a new microhabitat for plants. They differ in physicochemical properties from the surrounding soil, promoting pioneer (Semenov et al., 2001; Swihart, 1991; Van Staalduinen & Werger, 2007) and nitrophile plants (Ballová et al., 2019; La et al., 2003). Mounds in our study sites consisted mainly of stones larger than 2 cm, which make up most of the subsurface soil layers (Kabala et al., 2021). Fine soil particles, as well as soil moisture ejected by marmots from deeper layers, are probably removed by wind, like it has been found in desert environments (Sharma & Birla, 1975) long before it can be utilized by plants. Also, there is no evidence for the presence of deep organic matter in our study area (Kabala et al., 2021). Those are probably the reasons for the lack of plant succession on mounds we found, similar to another study in Eastern Pamir (Dotter, 2009). Furthermore, marmot activity causes a loss of inhabitable sites for plants, probably for decades. The total area of burrow mounds, based on an extrapolation of mounds covered by aerial photographs, is 0.94 ha. Burrow mounds cover between 0.06% and 0.35% of the high cluster and between 0.07% and 0.42% of the low cluster.

Researchers studying ecosystem engineering by burrowing animals have identified the crucial role of water availability in the interaction between those animals and plants. In arid regions, burrowing animals and the structures they form have a larger impact on plant species richness (Romero et al., 2015), soil nutrients (Decker, Eldridge, et al., 2019; Mallen‐Cooper et al., 2019), decomposition rate (Decker, Leonard, et al., 2019) as compared to more humid environments. However, these comparisons may be biased, as there are simply more burrowing animals and more studies on ecosystem engineers in arid regions (Coggan et al., 2018). Other authors found the effect size of burrow‐dwelling ecosystem engineers to be solely site‐ and species‐specific (Louw et al., 2019; Root‐Bernstein & Ebensperger, 2013), and our results seem to fit this description. In humid, productive ecosystems where plants are mainly limited by biotic factors, such as herbivory and competition, the effect of ecosystem engineering is small (Crain & Bertness, 2006). In arid ecosystems, burrowing animals enhance nutrient cycling, which is hampered by water shortage, thus locally increasing productivity (Gharajehdaghipour et al., 2016; Yu et al., 2017). However, below a certain level of water availability, as drought directly limits plant growth, this effect decreases as it probably occurred in this study. Our results do not undermine the paradigm on the relationship between abiotic factors and the effect size of ecosystem engineers, but they lengthen the abiotic gradient by extreme habitats, which are strongly understudied.

Eastern Pamir, similarly to other mountain regions, is particularly at risk due to climate change (Kohler et al., 2010). As temperatures are increasing, melting glaciers will continue to reveal patches of bare soil (Mętrak et al., 2015). But as precipitation in Eastern Pamir is expected to decrease even further (Lioubimtseva & Henebry, 2009; Normatov & Normatov, 2020), the succession of these areas will probably remain extremely slow and the impact of marmots on plants minimal. Rising temperatures may increase the rate of mineral weathering (Gislason et al., 2009), further diminishing the role of animal‐derived nutrients for plants.

Some of the studied plant groups belong to the functional group of cushion plants (*Acantholimon* sp., *Androsace* sp., and *Oxytropis* sp.), which are ecosystem engineers themselves. They were shown to promote the growth of other plants in the Mountains of Central Asia by facilitating soil formation and by storing nutrients and water (Wang et al., 2021; Yang et al., 2010). An interesting question that emerges from our study is the mutual relationship between the effects of both ecosystem engineers (cushion plants and marmots), as they could hypothetically strengthen or hamper each other's impact.

Despite the relatively small impact on plant nutritional status and vegetation cover, long‐tailed marmots inhabiting extremely dry mountain habitats, most probably have a crucial impact on the ecosystem. Given the burrow density, marmots are probably the most abundant animal species in terms of biomass. This can make them an important food source for predators inhabiting our study area: wolves (*Canis lupus*) red foxes (*Vulpes vulpes*), and snow leopards (*Uncia uncia*), which are known to prey on marmots, especially the two latter species (Blumstein & Robertson, 1995; Jumabay‐Uulu et al., 2014; Khatoon et al., 2017). Marmot burrows can provide unique refuges of stable temperature and high humidity, crucial for the development of insects, especially in arid environments (Pike & Mitchell, 2013; Whitford & Kay, 1999). Marmot burrows can be used by other animals as the only shelter from strong winds and predators. Due to the unique character of the studied environment, the mechanisms described in this paragraph, similar to our results on animal–plant interactions, can happen differently than in grasslands, steppes, and mountain meadows. We think that a lot of questions in this area are open to further research.

## AUTHOR CONTRIBUTIONS

**Piotr Chibowski:** Conceptualization (equal); data curation (equal); formal analysis (lead); investigation (equal); methodology (equal); visualization (lead); writing – original draft (lead); writing – review and editing (equal). **Marcin Zegarek:** Conceptualization (equal); data curation (equal); formal analysis (supporting); visualization (supporting); writing – review and editing (equal). **Aleksandra Zarzycka:** Conceptualization (equal); investigation (equal); methodology (equal); writing – review and editing (equal). **Małgorzata Suska‐Malawska:** Conceptualization (equal); funding acquisition (lead); investigation (supporting); methodology (equal); resources (lead); writing – review and editing (equal).

## CONFLICT OF INTEREST STATEMENT

The authors declare that they have no competing interests.

## Supporting information


Appendix S1.

Table S1.

## Data Availability

The data that support the findings of this study are openly available in RepOD at http://doi.org/10.18150/F8VYMX.
