# Supplementary material for: Ecosystem engineers in the extreme: The modest impact of marmots on vegetation cover and plant nitrogen and phosphorus content in a cold, extremely arid mountain environment
Source: Ecol Evol. 2023 Mar 27;13(3):e9948. doi: 10.1002/ece3.9948 (PMC10041373; doi:10.1002/ece3.9948)
Supplement: Supplementary file 1 — Appendix S1. [file ECE3-13-e9948-s001.docx]

Appendix S1. Methodological rationale

We are aware, that we used a sampling strategy different than in most studies of this type. Most of these differences were however caused by the unique and unstudied character of our study area. The most common approach to studying the impact of burrowing animals on plant nutrients is to compare those parameters of plants from areas which were affected and unaffected by the activity of animals (e.g. Clark et al., 2016; Louw et al., 2019). This is mainly done by visual inspection: the area around the burrow where the plant community is visibly different and plants have signs of herbivory is considered affected, while the surrounding area, which does not differ from sites without burrows is considered unaffected. In our study a rea, signs of herbivory where very sparse, and there was no visible increase in plant size of cover around the burrows. The whole study area, including the two terraces where the burrow cluster were located and where control plots would be established in this type of sampling, were very heterogeneous in terms of plant cover, species composition and possibly in nutrient status, especially around the high cluster (Kabala et al., 2021). Thus, establishing control plots would be difficult and susceptible to bias. Therefore, we sampled plants for biomass N and P analysis in a distance gradient and used regression to test the relationship, instead of pairwise comparison.

Using camera-equipped unmanned aerial vehicles is a growing trend in environmental studies (Anderson & Gaston, 2013). It has been previously used in studies similar to ours. Aerial photographs were used to locate burrows (Wang & Hou, 2021) as well as changes in plant cover caused by the activity of burrowing mammals (Qin, Sun, et al., 2020; Qin, Yi, et al., 2020). In our study it proved useful for verifying the burrow survey and to measure the area of burrow mounds. The limitations of our approach showed during the initial inspections of the captured photographs. The presence of clouds in all days of fieldwork caused constant changes in lightning conditions. Therefore, automatic mapping of vegetation patches based on pixel colour was impossible, and all analysis had to be done, or at least verified by hand. We limited the analysis to 20 m buffers around burrow entrances - mapping the whole area would be extremely time consuming. Not all vegetation could be seen on the images, as most plant species grow only in-between polygons shaped by frost (Kabala et al., 2021), mainly as single individuals and their colour is greyish-green. In turn, patches of cushion plants (*Acantholimon* sp., *Androsace* sp. and *Oxytropis* sp.) were clearly visible. Therefore, we decided to use cushion plants as a proxy for all vegetation. The most information on the relationship between plant and burrow distribution can be acquired from visual inspection of the aerial image with enhanced green channel (Fig 1B). Burrows do not seem to be preferably located on patches of denser vegetation. The majority of the largest vegetation patch is free of burrows. At the same time, several burrows (e.g. burrows which are furthest South) are located in areas with almost no vegetation. Unfortunately, we have no knowledge about the way marmots choose burrowing areas (apart from the obvious preference to build burrows on the edge of the terrace) which could help us understand the burrow distribution.

A decrease in vegetation cover due to burrowing animal activity is mostly caused by the presence of mounds close to the burrow entrance, which have not been colonised by plants (Dotter, 2009; Louw et al., 2019). Our results showed no possibility of plant regrowth on mounds in the present conditions in the studied area. Therefore, we excluded mounds from the analysis of vegetation cover and tried to check whether marmots modify plant recruitment on inhabitable soil.

Reviewers suggested, that many papers are incomparable to our study, as they describe the plants occurring on the different microhabitats created by burrowing mammals and not the burrow itself. We could not observe the presence of microhabitats around the burrows we studied. However, articles we refer to (Dotter, 2009; Louw et al., 2019) analyse the impact of nutrient input via excretion and of herbivory on plants, which is the same mechanism we aimed to study. While acknowledging the limitations of our study, we feel that the lack of studies of this type in our study area somehow justifies the lack of precise hypotheses and makes our results valuable for other researchers. Especially given the difficult logistics required to conduct studies in Tajikistan.

Bibliography (articles mentioned in Supporting Information 1, but not in the main text):

Anderson, K., & Gaston, K. J. (2013). Lightweight unmanned aerial vehicles will revolutionize spatial ecology. *Frontiers in Ecology and the Environment*, *11*(3), 138–146. https://doi.org/10.1890/120150

Clark, K. L., Branch, L. C., Hierro, J. L., & Villarreal, D. (2016). Burrowing herbivores alter soil carbon and nitrogen dynamics in a semi-arid ecosystem, Argentina. *Soil Biology and Biochemistry*, *103*, 253–261. https://doi.org/10.1016/j.soilbio.2016.08.027

Qin, Y., Sun, Y., Zhang, W., Qin, Y., Chen, J., Wang, Z., & Zhou, Z. (2020). Species monitoring using unmanned aerial vehicle to reveal the ecological role of Plateau pika in maintaining vegetation diversity on the northeastern Qinghai-Tibetan Plateau. *Remote Sensing*, *12*(15). https://doi.org/10.3390/RS12152480

Qin, Y., Yi, S., Ding, Y., Qin, Y., Zhang, W., Sun, Y., Hou, X., Yu, H., Meng, B., Zhang, H., Chen, J., & Wang, Z. (2020). Effects of plateau pikas’ foraging and burrowing activities on vegetation biomass and soil organic carbon of alpine grasslands. *Plant and Soil*. https://doi.org/10.1007/s11104-020-04489-1

Wang, S. L., & Hou, F. J. (2021). Burrow characteristics and ecological significance of Marmota himalayana in the northeastern Qinghai-Tibetan Plateau. *Ecology and Evolution*, *11*(13), 9100–9109. https://doi.org/10.1002/ece3.7754
