# Supplementary material for: Ecosystem engineers in the extreme: The modest impact of marmots on vegetation cover and plant nitrogen and phosphorus content in a cold, extremely arid mountain environment
Source: Ecol Evol. 2023 Mar 27;13(3):e9948. doi: 10.1002/ece3.9948 (PMC10041373; doi:10.1002/ece3.9948)
Supplement: Supplementary file 2 — Table S1. [file ECE3-13-e9948-s002.docx]

Table S1. Comparison of nutrient content and stable isotope ratios between the low and high cluster within plant species that were sampled in both clusters. Pairs that are bold differ significantly (p<0.05). TS – test statistic value, t for *t*-test, W for Wilcoxon test. * - Welch's *t*-test for unequal variances

| Species | Cluster | N | Total N (%) | Total P (%) | N:P | δ^15^N (‰) |
| --- | --- | --- | --- | --- | --- | --- |
| *Acantholimon* sp | High | 19 | 2.68 ± 0.32 | 0.1 ± 0.03 | **28.21 ± 27.92** | 1.07 ± 1.98 |
|  | Low | 14 | 2.74 ± 0.3 | 0.08 ± 0.03 | **38.23 ± 39.89** | 1.2 ± 1.27 |
|  |  | TS | t = 0.52 | t = 1.73 | t = -2.28 | t = -0.21 |
|  |  | df | 31 | 31 | 16.64 | 31 |
|  |  | p | >0.05 | >0.05 | <0.05* | >0.05 |
| *B. pamirica* | High | 6 | **4.48 ± 0.37** | 0.22 ± 0.02 | 20.27 ± 19.79 | 0.57 ± 1.1 |
|  | Low | 6 | **3.21 ± 0.57** | 0.22 ± 0.1 | 16.81 ± 16.4 | 1.51 ± 2.51 |
|  |  | TS | t = 4.18 | W = 29 | t = 0.26 | t = -0.79 |
|  |  | df | 10 | - | 5.89 | 10 |
|  |  | p | <0.05 | >0.05 | >0.05* | >0.05 |
| *Oxytropis* sp | High | 31 | **3.83 ± 0.55** | **0.19 ± 0.03** | **20.75 ± 21.97** | -0.97 ± 1.27 |
|  | Low | 15 | **3.38 ± 0.38** | **0.14 ± 0.02** | **24.67 ± 25.86** | -1.08 ± 1.11 |
|  |  | TS | t = 2.80 | t = 5.66 | t = -4.02 | W = 272 |
|  |  | df | 44 | 44 | 44 | - |
|  |  | p | <0.05 | <0.0001 | <0.001 | >0.05 |
| *L. eriocalyx* | High | 10 | 4.62 ± 0.39 | 0.18 ± 0.04 | 26.9 ± 24.84 | **2.44 ± 2.07** |
|  | Low | 10 | 4.26 ± 0.58 | 0.19 ± 0.08 | 25.17 ± 26.77 | **-0.35 ± 1.67** |
|  |  | TS | W = 72 | W = 50 | t = 0.57 | t = 4.74 |
|  |  | df | - | - | 18 | 18 |
|  |  | p | >0.05 | >0.05 | >0.05 | <0.001 |
